# NOP Receptor Agonist Ro 64-6198 Decreases Escalation of Cocaine Self-Administration in Rats Genetically Selected for Alcohol Preference

**DOI:** 10.3389/fpsyt.2019.00176

**Published:** 2019-03-29

**Authors:** Hongwu Li, Giulia Scuppa, Qianwei Shen, Alessio Masi, Cinzia Nasuti, Nazzareno Cannella, Roberto Ciccocioppo

**Affiliations:** ^1^College of Chemical Engineering, Changchun University of Technology, Changchun, China; ^2^Pharmacology Unit, School of Pharmacy, University of Camerino, Camerino, Italy

**Keywords:** abuse, addiction, psychostimulants, drug-seeking, opioids

## Abstract

Cocaine dependence is a psychiatric condition for which effective medications are still lacking. Published data indicate that an increase in nociceptin/orphanin FQ (N/OFQ) transmission by NOP receptor activation attenuates cocaine-induced place conditioning and the locomotor sensitization effects of cocaine. This suggests that the activation of the N/OFQ receptor (NOP) may attenuate the motivation for psychostimulants. To further explore this possibility, we investigated the effect of the potent and selective NOP receptor agonist Ro 64-6198 on cocaine intake under 1 h short access (ShA) and 6 h long access (LgA) operant self-administration conditions in rats. We used Marchigian Sardinian alcohol-preferring (msP) rats and Wistar control rats. msP rats were used because we recently found that this rat line, originally selected for excessive alcohol drinking and preference, exhibits a greater propensity to escalate cocaine self-administration following LgA training. msP rats are also characterized by innate overexpression of the N/OFQ-NOP system compared with Wistar rats. Wistar and msP rats both exhibited an increase in cocaine self-administration under LgA conditions, with a higher trend toward escalation in msP rats. In Wistar rats, the intraperitoneal administration of Ro 64-6198 (0. 1 and 3 mg/kg) significantly decreased ShA cocaine self-administration. In Wistar rats that underwent LgA cocaine self-administration training, Ro 64-6198 induced no significant effect either during the first hour of self-administration or after the entire 6 h session. In msP rats, Ro 64-6198 significantly reduced cocaine self-administration both under ShA conditions and in the first hour of the LgA session. At the end of the 6 h session, the effect of Ro 64-6198 was no longer observed in msP rats. The highest dose of Ro 64-6198 (3 mg/kg) did not affect saccharin self-administration in msP rats but reduced saccharin self-administration in Wistar rats. Altogether, these data suggest that NOP receptor activation attenuates cocaine self-administration, and this effect tends to be more pronounced in a rat line with innately higher NOP receptor expression and that more robustly escalates cocaine intake.

## Introduction

Cocaine is the most commonly abusedillicit psychostimulant, and its use is linked to serious physical, psychiatric, socioeconomic, and legal problems (1). Effective medications for the treatment of cocaine addiction are lacking. The development of medications that can control cocaine intake and seeking would represent a significant medical breakthrough.

Cocaine is often co-abused with alcohol. Cocaine dependence and alcohol dependence share several genetic traits, indicating that common predisposing factors may exist (2–4). We recently found that Marchigian Sardinian alcohol-preferring (msP) rats, which are genetically selected for excessive alcohol drinking and preference, also exhibit neurophysiological and pharmacological traits that confer a predisposition to psychostimulant abuse (5). We recently found that msP rats exhibited alterations of functional magnetic resonance imaging activity and an increase in nucleus accumbens dopamine release in response to an amphetamine challenge compared with Wistar rats. msP rats also exhibited a higher propensity to escalate cocaine intake under extended access (6 h/day) self-administration conditions (5). Compared with heterogeneous stock Wistars rats (i.e., the rat strain from which msP rats originate), the msP line appears to present an addiction-prone phenotype. msP rats are also characterized by inherited neurophysiological adaptations of several neurotransmitter systems that may contribute to their vulnerable phenotype (6–8). One such system consists of nociceptin/orphanin-FQ (N/OFQ) peptide and its NOP receptor, known for being structurally similar to dynorphin A and kappa opioid, respectively (9, 10). The activation of NOP receptors has been shown to attenuate the motivation for various drugs of abuse (11–18).

Intracranial N/OFQ administration inhibited psychostimulant-induced conditioned place preference and locomotor sensitization in rats (19, 20). NOP receptor knockout mice exhibited higher cocaine-induced conditioned place preference compared with their wildtype counterparts (21). Additionally, NOP knockout mice showed increased psychomotor sensitization to cocaine (22) and N/OFQ abolished cocaine-induced psychomotor sensitization in wildtype but not NOP knockout mice (23). Importantly, intracerebroventricular N/OFQ administration did not induce conditioned place preference or aversion, suggesting that NOP receptor agonists do not have motivational properties *per se* (24, 25). Although data on the effect of NOP receptor activation on cocaine-related behaviors have been published, direct evidence of an effect of NOP receptor agonists on cocaine self-administration are limited to buprenorphine and cebranopadol, two molecules that simultaneously activate NOP and μ opioid receptors (16–18). The present study investigated the effect of the potent and selective NOP receptor agonist Ro 646198 on cocaine intake in rats that were exposed to a daily 1 h short access (ShA) or 6 h long access (LgA) operant self-administration sessions. The study was performed in heterogeneous Wistar and msP rats. msP rats exhibit overexpression of the N/OFQ system and a greater propensity to escalate cocaine self-administration. msP rats also exhibit high sensitivity to NOP receptor agonists, and a history of dependence enhances NOP expression. Therefore, we predicted that Ro 64-6198 would be more effective in rats that were exposed to LgA cocaine self-administration and that msP rats would be more sensitive to Ro 646198 than Wistar rats (8, 26).

## Materials and Methods

### Animals

The experiments were conducted with male Wistar rats (Charles River, Calco, Italy) and msP rats (bred at the School of Pharmacy, University of Camerino, Italy). The rats weighed 200–250 g at the beginning of the study. They were housed in pairs in a room under a reverse 12 h/12 h light/dark cycle (lights off at 9:00 a.m.) with constant temperature (20–22°C) and humidity (45–55%). Food and water were provided *ad libitum*. The animals were treated in accordance with the guidelines of the European Community Council Directive for Care and Use of Laboratory Animals. The experimental procedures were approved by the Italian Ministry of Health (authorization no. 414/2016-PR).

### Drugs

Cocaine hydrochloride (Sigma, St. Louis, MO, USA) was dissolved in sterile saline. Saccharin (Sigma, Italy) was dissolved in tap water. The NOP receptor agonist Ro 64-6198 was dissolved in 10% dimethylsulfoxide, 10% Tween-80, and 80% water. Doses timing and route of administration of Ro 64-6198 were chosen based on earlier NOP binding studies indicating that an acute IP injection of 3.2 mg/kg of Ro 64-6198 induced maximal NOP receptor occupancy after 30 min. Good receptor occupancy was maintained for approximately 3 h (27).

### Catheter Implantation

The rats were anesthetized by an intramuscular injection of 100–150 μl of a solution that contained tiletamine chlorhydrate (58.17 mg/ml) and zolazepam chlorhydrate (57.5 mg/ml). For intravenous surgery, incisions were made to expose the right jugular vein. A catheter that was constructed from micro-renathane tubing (inner diameter = 0.020 inches, outer diameter = 0.037 inches) was subcutaneously positioned between the vein and back. After insertion into the vein, the proximal end of the catheter was anchored to the muscles that underlie the vein with surgical silk. The distal end of the catheter was attached to a stainless-steel cannula that was bent at a 90° angle. The cannula was inserted into a support that consisted of dental cement on the back of the animals and was covered with a plastic cap. Immediately after surgery, the rats were treated intramuscularly with 200 μl of enrofloxacin (50 mg/ml, Baytril, Germany).

The rats were allowed to recover for 1 week before self-administration training. Catheters patency was confirmed by an intravenous injection of 150 μl of sodium pentothal (25 mg/ml, Intervet, Italy). Before each self-administration session, the catheters were flushed with 100 μl of heparinized saline (20 UI/ml) that contained 0.5 mg/ml enrofloxacin.

### Self-Administration Apparatus

The self-administration stations consisted of operant conditioning chambers (Med Associates, USA) that were enclosed in sound-attenuating, ventilated environmental cubicles. Each chamber was equipped with two retractable levers that were located in the front panel of the chamber. Cocaine was delivered intravenously through a plastic tube that was connected to an infusion pump. Saccharin was delivered in a receptacle that was connected to the infusion pump and located between the two levers. Responses on the right (active) lever activated the infusion pump, and responses on the left (inactive) lever were recorded but did not have any programmed consequences. In both the cocaine and saccharin sessions, activation of the pump resulted in the delivery of 0.1 ml of fluid. A computer controlled the delivery of cocaine solution and recorded the behavioral data.

### General Cocaine Self-Administration Procedure

Wistar and msP rats were initially trained to self-administer cocaine (0.25 mg/infusion) under a fixed-ratio 1 (FR1) schedule of reinforcement for 1 h/day for 5 days. Afterward, the reinforcement contingency was switched to an FR5 schedule, and the rats of each line were split into two subgroups: self-administration in a 1 h short-access (ShA) session and self-administration in a 6 h long access (LgA) session. The LgA and ShA sessions under an FR5 schedule continued for 23 days, after which the effect of Ro 64-6198 on cocaine self-administration was tested.

### Effect of Ro 64-6198 on Cocaine Self-Administration in Wistar Rats

Starting from the 24th FR5 session, we tested the effect of Ro 64-6198 and its vehicle on cocaine self-administration in Wistar rats that were trained under ShA (*n* = 10) and LgA (*n* = 10) conditions. Using a within-subjects counterbalanced design, 30 min before the session, the rats received Ro 64-6198 (1.0 and 3.0 mg/kg, i.p.) or its vehicle. The tests were repeated every third day. On the first intervening day, the rats remained in their home cage. On the second intervening day, they underwent a baseline cocaine self-administration session. The number of infusions was recorded after the first hour of self-administration and also at 6 h in the LgA group.

### Effect of Ro 64-6198 on Cocaine Self-Administration in msP Rats

Similar to Wistar rats, starting from the 24th FR5 session, we tested the effect of Ro 64-6198 (1.0 and 3.0 mg/kg, i.p.) and its vehicle on cocaine self-administration in msP rats that were trained under ShA (*n* = 9) and LgA (*n* = 9) conditions. The tests were performed using a within-subjects counterbalanced design at intervals of 3 days. The number of infusions was recorded after the first hour of self-administration and also at 6 h in the LgA group.

### Effect of Ro 64-6198 on Saccharin Self-Administration in Wistar and msP Rats

Two additional groups of Wistar (*n* = 9) and msP (*n* = 9) rats were trained in daily 1 h saccharin self-administration sessions under an FR1 schedule of reinforcement. When the rats reached a stable baseline of saccharin intake, we tested the effect of Ro 64-6198 (1.0 and 3.0 mg/kg, i.p.) and its vehicle on saccharin self-administration. Thirty minutes before the sessions, the rats received Ro 64-6198 (1.0 and 3.0 mg/kg, i.p.) and its vehicle in a counterbalanced Latin-square design. The tests were repeated every third day.

### Statistical Analyses

The number of infusions that were received by ShA rats was compared with the number of infusions that were received by LgA rats during the first hour of the daily sessions using two-way repeated-measures analysis of variance (ANOVA), with session length (ShA *vs*. LgA) as the between-subjects factor and time (days) as the repeated measure. The escalation of cocaine self-administration was analyzed using one-way ANOVA, with time as the repeated measure. The effect of Ro 64-6198 on cocaine and saccharin self-administration was analyzed using one-way ANOVA, with dose as the within-subjects factor. Wistar and msP rats were analyzed separately. Significant main effects in the ANOVA were followed by the Newman-Keuls *post hoc* test for escalation and Dunnett's *post hoc* test for Ro 64-6198. Values of *p* < 0.05 were considered statistically significant.

## Results

### Escalation of Cocaine Self-Administration in Wistar and msP Rats

One LgA Wistar rat and one ShA Wistar rat became sick during training; therefore, only *n* = 9 LgA Wistar rats and *n* = 9 ShA Wistar rats were considered for the analyses. The escalation of cocaine intake reflects an increase in the number of infusions that are obtained daily over time. Escalation occurs during LgA sessions and usually is measured by analyzing the number of rewards that are earned during the first hour of intake in the LgA session compared with the ShA session (28).

**Figure 1:**
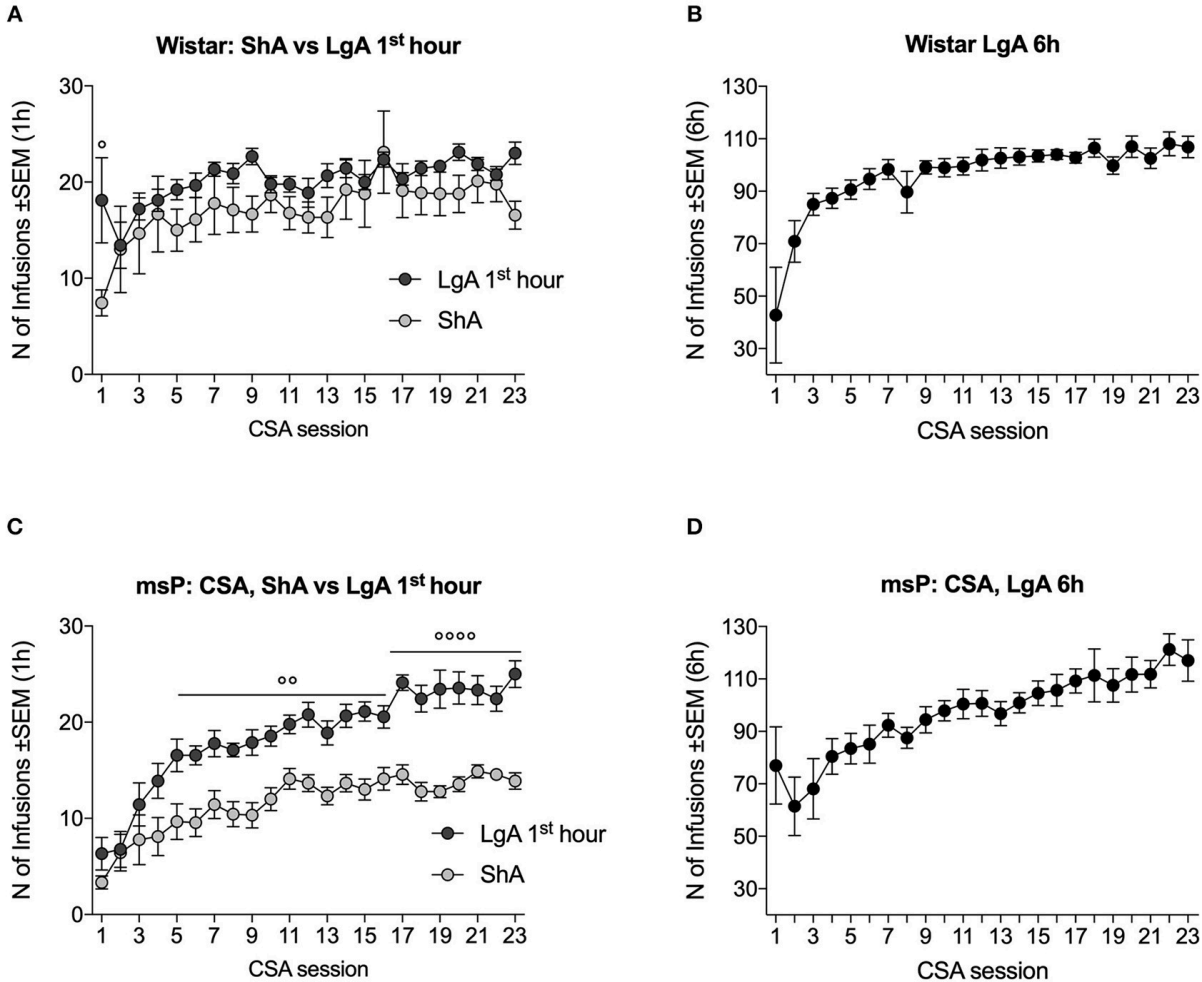
Escalation of cocaine self-administration (CSA) in Wistar and msP rats. **(A)** The number of cocaine infusions that were received by Wistar rats in the first hour of ShA and LgA sessions was similar throughout training, with the exception of the first day. **(B)** The number of infusions that were received by Wistar rats during the entire 6 h LgA session increased over time. **(C)** The number of infusions that were received by msP rats during the first hour of the daily LgA sessions increased over training and was higher than ShA-trained msP rats beginning in the fifth session. **(D)** The number of infusions that were received by LgA-trained msP rats increased over training during the entire 6 h session. The data are expressed as mean ± SEM. ° *p* < 0.05, °° *p* < 0.01, °°°° *p* < 0.001, compared with ShA.

In Wistar rats, the ANOVA indicated no effect of session length [*F*_(1, 16)_ = 1.5, *p* > 0.05] but a significant effect of time [*F*_(22, 352)_ = 6.5, *p* < 0.0001] and a significant session length × time interaction [*F*_(22, 352)_ = 1.7, *p* < 0.05]. The two groups differed in intake on the first day under the FR5 schedule but earned a similar number of infusions during the remainder of training (Figure 1A). LgA Wistar rats exhibited an increase in lever pressing over time [*F*_(8, 176)_ = 8.1, *p* < 0.01], with a progressive increase in the total number of daily (6 h) cocaine infusions that were earned (Figure 1B).

In msP rats, ANOVA was used to compare the number of infusions that were received in the ShA group and LgA group during the first hour of the session. The ANOVA indicated significant main effects of session length [*F*_(1, 16)_ = 40.9, *p* < 0.0001] and time [*F*_(22, 352)_ = 24.7, *p* < 0.0001] and a significant session length × time interaction [*F*_(22, 352)_ = 2.4, *p* < 0.001]. The *post hoc* analysis indicated that LgA rats exhibited an increase in the number of infusions over time and differed significantly from ShA rats starting from the fifth session (Figure 1C). LgA msP rats exhibited an increase in lever pressing over time [*F*_(8, 176)_ = 8.9, *p* < 0.001], with a progressive increase in the total number of daily (6 h) cocaine infusions that were earned (Figure 1D).

When the escalation ratio, calculated as a difference between the average infusions of the last 3 minus the first 3 LgA sessions, of msP and Wistar rats was compared, results indicated a significant difference between the two rat lines [*t*_16_ = 2.9, *p* < 0.05]. Escalation ratio was 39.3 ± 6.6 in msPs and 19.2 ± 3.3 in Wistars.

### Effect of Ro 64-6198 on Cocaine and Saccharin Self-Administration in Wistar Rats

**Figure 2:**
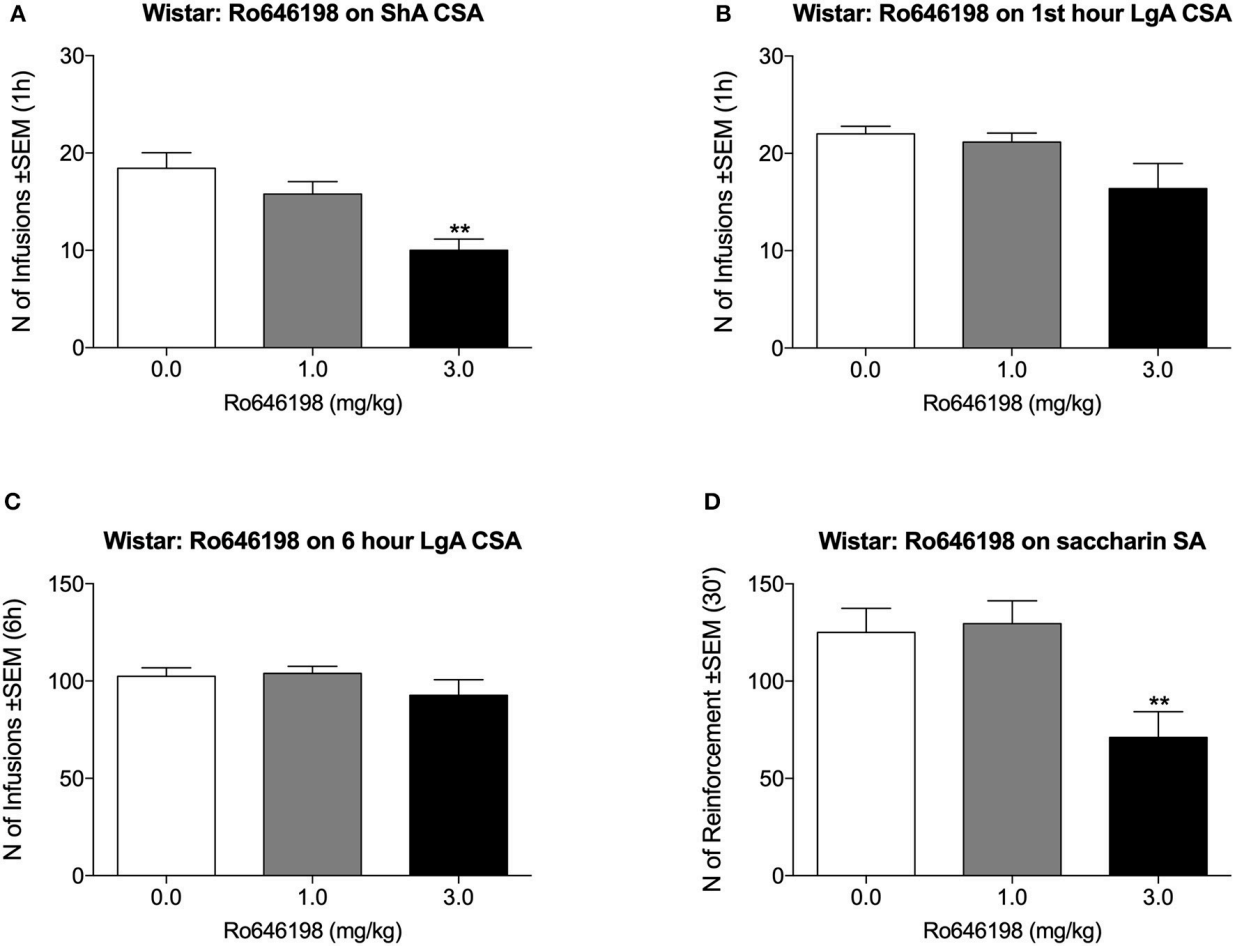
Effect of Ro 64-6198 on cocaine and saccharin intake in Wistar rats. **(A)** Ro 64-6198 at a dose of 3.0 mg/kg decreased the number of infusions that were received by ShA rats. **(B,C)** Ro 64-6198 did not affect cocaine self-administration in LgA-trained rats either during the first hour of the session **(B)** or during the entire 6 h session **(C)**. **(D)** Ro 64-6198 at a dose of 3.0 mg/kg decreased saccharin self-administration. The data are expressed as mean ± SEM. ^**^p < 0.01, compared with Ro 64-6198 vehicle (0.0 mg/kg).

We next tested the effect of Ro 64-6198 on cocaine self-administration in Wistar rats. One additional LgA Wistar rat was excluded from the analysis because of the loss of catheter patency. Ro 64-6198 significantly decreased the number of infusions that were received by ShA Wistar rats [*F*_(2, 8)_ = 16.5, *p* < 0.01]. Dunnett's *post hoc* test revealed that 3.0 mg/kg but not 1.0 mg/kg Ro 64-6198 significantly decreased the number of infusions compared with vehicle (*p* < 0.01; Figure 2A). Under LgA conditions, the ANOVA indicated no effect of treatment either during the first hour [*F*_(2, 7)_ = 2.8, *p* > 0.05] or during the entire 6 h session [*F*_(2, 7)_ = 2.0, *p* > 0.05; Figures 2B,C]. The ANOVA of the effect of Ro 64-6198 on saccharin self-administration indicated a main effect of treatment [*F*_(2, 8)_ = 27.7, *p* < 0.001]. Dunnett's *post hoc* test showed that 3 mg/kg Ro 64-6198 significantly decreased saccharin self-administration (*p* < 0.01; Figure 2D).

### Effect of Ro 64-6198 on Cocaine and Saccharin Self-Administration in msP Rats

**Figure 3:**
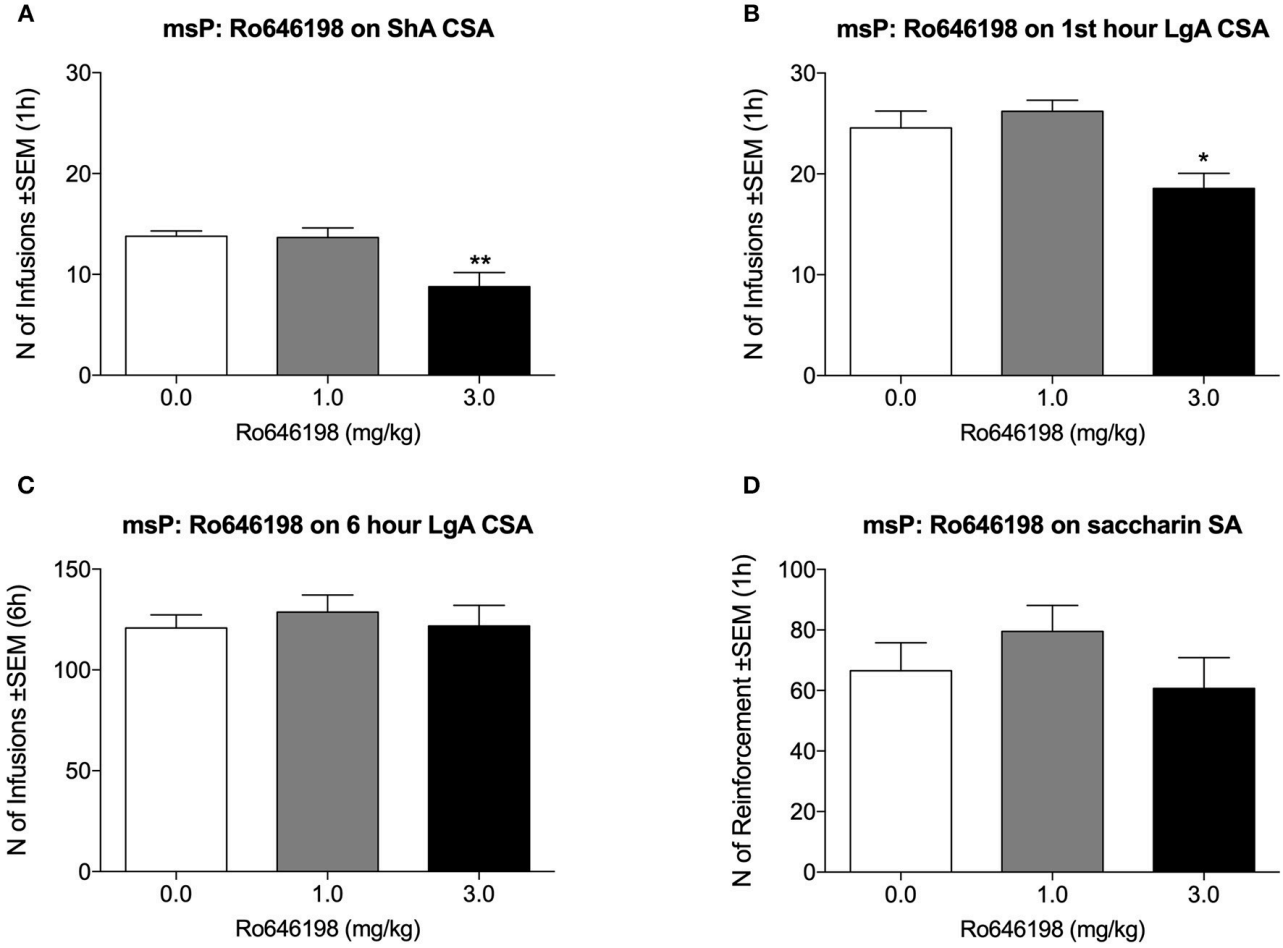
Effect of Ro 64-6198 on cocaine and saccharin intake in msP rats. **(A)** Ro 64-6198 at a dose of 3.0 mg/kg decreased the number of infusions that were received in the ShA session. **(B,C)** Ro 64-6198 at a dose of 3.0 mg/kg decreased the number of infusions that were received by LgA rats during the first hour of the session **(B)** but not during the entire 6 h session **(C)**. **(D)** Ro 64-6198 did not affect saccharin self-administration. The data are expressed as mean ± SEM. ^*^p < 0.05, ^**^p < 0.01, compared with Ro 64-6198 vehicle (0.0 mg/kg).

In msP rats, Ro 64-6198 significantly decreased cocaine self-administration both in the ShA condition [*F*_(2, 8)_ = 15.3, *p* < 0.001; Figure 3A] and in the first hour of the LgA condition [*F*_(2, 8)_ = 11.6, *p* < 0.01; Figure 3B]. Dunnett's *post hoc* test revealed a significant effect of 3.0 mg/kg Ro 64-6198 (*p* < 0.01). The ANOVA of the effect of Ro 64-6198 at 6 h in the LgA group revealed no effect of treatment [*F*_(2, 8)_ = 0.3, *p* > 0.05; Figure 3C]. The ANOVA indicated no effect of Ro 64-6198 on saccharin self-administration [*F*_(2, 8)_ = 2.0, *p* > 0.05; Figure 3D].

## Discussion

The present study found that cocaine intake was similar in Wistar and msP rats under ShA self-administration conditions, but Wistar rats received a slightly higher number of infusions. When the rats were exposed to LgA self-administration, msP rats exhibited greater escalation of cocaine intake. These results replicate our recent study, in which we found that msP rats exhibited greater escalation of cocaine self-administration compared with Wistar rats. Additionally, in response to an amphetamine challenge, msP rats exhibited greater locomotor stimulation, greater activation of mesolimbic circuitry, and higher extracellular levels of dopamine in the nucleus accumbens compared with Wistar rats (5). The msP rat line was originally selected for excessive alcohol drinking and preference. However, unknown is why they are also hypersensitive to psychostimulants. One possibility is that some of the genetic traits that confer greater vulnerability to psychostimulants also result in an increase in alcohol intake (i.e., common genetic factors may be responsible for increases in both alcohol consumption and the vulnerability to psychostimulants). In humans, cocaine and alcohol are often co-abused, and common genetic traits that confer vulnerability to their use have begun to emerge (2–4).

Previous studies showed that Sardinian alcohol-preferring rats, from which msP rats were derived, are characterized by the lower expression of dopamine D_1_ and D_2_ receptors in the striatum (29, 30). Under basal condition, these two rat lines may present a hypodopaminergic state that motivates them to take higher amounts of alcohol and cocaine. Theserat lines' ability to increase mesolimbic dopamine levels may help counteract such a deficiency in dopamine. Clinical studies have shown that human addicts have relatively low levels of dopamine receptors in the ventral striatum (31–34). Similar to msP rats, human addicts also present an increase in striatal activation, revealed by functional magnetic resonance imaging, in response to drug-related stimuli (5, 35–37). One possibility is that msP rats may represent an animal model that mimics conditions that are associated with advanced stages of the addiction cycle, reflected by their greater tendency to escalate drug use. Another neurochemical alteration that has been detected in msP rats is overexpression of the N/OFQ system in various mesolimbic structures (8). The activation of NOP receptors following the administration of N/OFQ in the ventral tegmental area attenuated dopamine release in the nucleus accumbens (38). The intracerebroventricular administration of N/OFQ suppressed the morphine-induced increase in extracellular dopamine levels in the nucleus accumbens (39). Moreover, N/OFQ administration in the nucleus accumbens attenuated the ability of cocaine to enhance local extracellular dopamine levels (40). These data suggest that greater activity of the N/OFQ system may further contribute to the reduction of the basal tone of the dopamine system, thus contributing to the motivation to consume drugs of abuse. This possibility is indirectly supported by a previous study, in which NOP receptor knockout rats self-administered less cocaine, alcohol, and heroin compared with wildtype controls (41). Refuting this hypothesis, however, is evidence that the pharmacological activation of NOP receptors attenuates the motivation for several drugs of abuse, including cocaine and amphetamine (19, 20, 42–45). To clarify the role of the N/OFQ system in the modulation of drug abuse-related behaviors, we tested the effect of the selective and potent NOP receptor agonist Ro 64-6198 on cocaine self-administration in both Wistar and msP rats. As expected, Ro 64-6198 reduced ShA cocaine self-administration in both Wistar and msP rats. Under LgA conditions, the effect of Ro 64-6198 was significant only in msP rats. Moreover, the half-life of Ro 64-6198 in rodents is relatively long (5.5 h), and the significant effect of Ro 64-6198 that was observed in the first hour of the LgA session was not observed at 6 h (27).

In Wistar rats, 3 mg/kg Ro 64-6198 significantly decreased saccharin self-administration. This effect was not observed in msP rats. This may suggest that the effect of Ro 64-6198 on self-administration is secondary to the nonspecific inhibition of locomotor activity. However, this possibility is unlikely. Although Ro 64-6198 reduced saccharin self-administration in Wistar rats, it did not affect saccharin self-administration in msP rats. Previous studies reported that Ro 64-6198 doses up to 3 mg/kg exert specific effects that are not linked to motor impairment (46, 47).

Previous studies also showed that NOP receptor activation by Ro 64-6198 reduced the motivation for alcohol and morphine. Therefore, the lower motivation to pursue a reward may also extend to natural reinforcers (48–50). This hypothesis is supported by previous findings, in which the stimulation of N/OFQ transmission in NOP receptor knockout mice suppressed both basal and drug-induced increases in the hedonic state (51).

The activation of NOP receptors leads to rapid and prolonged receptor desensitization (52, 53). For example, after treatment with Ro 64-6198, NOP receptors rapidly internalized, and N/OFQ-mediated transmission remained impaired for at least 30 min (27). Based on these findings, one hypothesis is that the effect on cocaine self-administration may be mediated by NOP receptor desensitization rather than NOP receptor activation. This may explain why Ro 64-6198 was slightly more effective in msP rats than in Wistar rats because of msP rats' innate overexpression of NOP receptors that may be more sensitive to desensitization. This receptor desensitization hypothesis could also explain why the effect of Ro 64-6198 is relatively short (1 h), notwithstanding its relatively long half-life of 5.5 h. After 1 h, the desensitized NOP receptors may progressively become available again. Finally, the receptor desensitization hypothesis may reconcile recent findings that NOP-deficient rats exhibited lower motivation for cocaine and self-administered less cocaine (41) and may explain why, similar to agonist, NOP antagonists could reduce drug self-administration (54, 55).

Consistent with the desensitization hypothesis, it is tempting to hypothesize that chronic treatment with Ro 64-6198 would have led to a more pronounced effect, possibly leading to reduction of cocaine intake also in Wistar rats. Earlier studies, however, have shown that chronic (25 days) and acute treatment with Ro64-6198 produced comparable reduction of NOP binding levels due to receptor internalization (27). Based on this observation we speculate that our treatment condition was sufficient to detect an effect of Ro 64-6198. To further asses this conclusion future studies will have to investigate the effect of chronic drug treatment on cocaine self-administration. Another potential limitation is that the present study was limited to the analysis of the effect of Ro 64-6198 on cocaine intake without exploring its action of the motivation for the drug using progressive ratio, punished responding or second order schedules contingencies. The absence of these data hamper the possibility to drive clear conclusions on the effect of NOP activation on the motivation of this psychostimulant. Finally, we did not explore whether LgA exposure to cocaine may have led to a dependent-like state signaled by expression of drug withdrawal or hyper-anxiety. However, these conditions were demonstrated in earlier studies in which the same LgA schedule used here was applied (56).

In conclusion, the present study found that msP rats escalated their cocaine self-administration more rapidly than Wistar rats, and NOP receptor activation by Ro 64-6198 reduced cocaine consumption. The mechanism by which NOP receptor agonism leads to such effects is unknown, but receptor desensitization may be one possibility.

## Data Availability

The raw data supporting the conclusions of this manuscript will be made available by the authors, without undue reservation, to any qualified researcher.

## Author Contributions

RC was responsible for the study concept and design. HL, GS, and QS performed the experiments. CN and AM assisted with the data analysis and interpretation of findings. HL, AM, NC, and RC drafted the manuscript. All of the authors critically reviewed the content and approved the final version for publication.

### Conflict of Interest Statement

The authors declare that the research was conducted in the absence of any commercial or financial relationships that could be construed as a potential conflict of interest.
